# Oxidative Stress and Arginine/Nitric Oxide Pathway in Red Blood Cells Derived from Patients with Prediabetes

**DOI:** 10.3390/biomedicines10061407

**Published:** 2022-06-14

**Authors:** Sonia Eligini, Benedetta Porro, José Pablo Werba, Nicolò Capra, Stefano Genovese, Arianna Greco, Viviana Cavalca, Cristina Banfi

**Affiliations:** 1 Centro Cardiologico Monzino, IRCCS, 20138 Milano, Italy; sonia.eligini@cardiologicomonzino.it (S.E.); benedetta.porro@cardiologicomonzino.it (B.P.); nicolo.capra@cardiologicomonzino.it (N.C.); stefano.genovese@cardiologicomonzino.it (S.G.); arianna.greco@cardiologicomonzino.it (A.G.); cristina.banfi@cardiologicomonzino.it (C.B.); 2 Dipartimento di Scienze Cliniche e di Comunità, Università Degli Studi di Milano, 20122 Milano, Italy; viviana.cavalca@unimi.it

**Keywords:** prediabetes, oral glucose tolerance test, red blood cells, nitric oxide pathway, eryptosis

## Abstract

The effects of the oral glucose tolerance test (OGTT) on red blood cells (RBCs) have not been thoroughly investigated, although it is known that the ingestion of 75 g of glucose during OGTT results in a systemic state of inflammation and oxidative stress. Therefore, we evaluated the effect of OGTT on oxidative stress and L-arginine/Nitric Oxide (L-Arg/NO) metabolic pathway in RBCs obtained from patients with prediabetes. Blood samples were collected from all participants before (T0) and at 10 (T1), 20 (T2), 30 (T3), 60 (T4), 90 (T5), 120 (T6), 150 (T7), and 180 (T8) minutes after glucose loading. Results showed a significant increase in oxidative stress status characterized by a rise in the GSSG/GSH ratio at T4 and T6 that increased in parallel with a reduction of NO production in RBCs. In addition, in this time frame, increased exposure of phosphatidylserine on RBCs membrane was observed. These metabolic modifications were rescued at T8, together with an increase in activated RBC NO synthase expression. These findings provide a possible explanation of the phenomena occurring after glucose loading and suggest that, even in the early stages of diabetes, it may be important to avoid acute variations in glycemia in order to prevent diabetic complications.

## 1. Introduction

Type 2 diabetes mellitus is preceded by a clinical condition, commonly called “prediabetes”, which is characterized by an absence of symptoms. In this phase two major indicators of imbalance in glucose metabolism can be identified: impaired glucose tolerance (IGT) and impaired fasting glucose (IFG) [1,2]. IFG and IGT conditions do not represent a disease, but risk factors for type 2 diabetes [3,4,5,6] and cardiovascular disease [7,8,9,10]; therefore, it is important in individuals with prediabetes to search for other risk factors for diabetes and cardiovascular disease.

IFG and IGT are identified by measurements of fasting plasma glucose and by 2-h plasma glucose levels during 75-g oral glucose tolerance test (OGTT) [11]. The presence of IFG and IGT increases the risk of developing type 2 diabetes by 3- to 10-fold [12], with an estimate that approximately 70% of people with prediabetes will develop diabetes [13]. Observational studies indicate that prediabetes is often associated with several conditions commonly present in overt diabetes. These include myocardial infarction, stroke, peripheral arterial disease, and also microvascular damages which may evolve to retinopathy, neuropathy, and nephropathy [14]. The mechanisms by which prediabetes may increase the risk of diabetes and vascular complications are still not fully known. Several mechanisms have been proposed, and among them, oxidative stress, mainly mediated by hyperglycemia-induced generation of free radicals [15]. Indeed, in healthy subjects with normal glucose tolerance, as well as in patients with IGT or diabetes, acute hyperglycemia markedly increases oxidative stress and reduces the plasma antioxidant capacity [16,17]. Oxidative stress [18] or other cell stressors, such as energy depletion [19], calcium entry [20], and osmotic shock [21], triggers eryptosis, the suicidal erythrocyte death characterized by cell shrinkage, membrane blebbing, and cell membrane scrambling with phosphatidylserine (PS) translocation from the inner leaflet of the membrane to the erythrocyte surface [20]. In line, the exposure of red blood cells (RBCs) to high levels of glucose induces cell membrane scrambling with subsequent PS exposure at the cell surface [22]. Accordingly, eryptosis is increased in diabetes [23,24], a condition wherein it might contribute to the shortened life span of erythrocytes [24] and their fragility [23]. In addition, the exposure of PS at the surface of erythrocytes promotes the adhesion of eryptotic cells to the vascular wall, compromising the blood flow in the microcirculation [25], and stimulating thrombin generation and fibrin formation [26]. Thus, an increased eryptosis might contribute to enhance thrombotic risk [27]. In contrast, eryptosis is inhibited by nitric oxide (NO) [28] and erythropoietin [29]. Indeed, it has been shown that the NO levels are reduced during an OGTT compared with fasting [30], leading to endothelial dysfunction [31]. It is known that RBCs are able to produce NO as they express a functional NO-synthase, so they may be considered as an important additional source of NO for the maintenance of vascular homeostasis [32,33,34]. However, there is currently a lack of information about the L-arginine (Arg)/NO metabolic pathway in RBCs after a glucose load. Thus, in the present study, we investigated oxidative stress, the L-Arg/NO metabolic pathway, and the PS exposure in RBCs isolated from prediabetic patients during a 75-g OGTT. 

## 2. Materials and Methods

### 2.1. Study Population

The study was carried out in accordance with the Declaration of Helsinki after local Research Ethics Committee approval (n° CCFM S151/210). Written informed consent was obtained from all participants. Eighteen consecutive patients (age range 50–75 years) with a diagnosis of prediabetes, based on the American Diabetes Association criteria [11], with IFG levels between 5.6 and 6.9 mmol/L were enrolled at Centro Cardiologico Monzino IRCCS. The exclusion criteria included diabetes (by either fasting plasma glucose levels or postprandial glycemia during 75-g OGTT), positive history of stroke, coronary heart disease, neoplastic disease, and inflammatory disease.

### 2.2. Blood Collection

EDTA-anticoagulated blood was drawn from the antecubital vein of subjects while fasting, to obtain whole blood (WB), plasma, and RBCs samples. After centrifugation, plasma was separated and stored at −80 °C until analysis. Serum was obtained from blood clotted for 2 h at 37 °C and serum sample were stored at −80°C until analysis. Aliquots of packed RBCs were immediately lysed by cold deionized water (1:1, *v*:*v*) and stored at −80 °C, until analysis. For the evaluation of NO-synthase (NOS), RBCs were washed three times in phosphate-buffered saline (PBS).

### 2.3. Oral Glucose Tolerance Test

Patients underwent OGTT between 7.00 and 9.00 am, according to WHO recommendations. After an overnight fast, a standard 75-g oral dose of glucose was administered immediately after the basal blood draw (T0). Subsequent blood samples were collected 10 (T1), 20 (T2), 30 (T3), 60 (T4), 90 (T5), 120 (T6), 150 (T7), and 180 (T8) minutes after glucose loading. 

### 2.4. Insulin Resistance and β-Cell Function Evaluation

The homeostatic model assessment (HOMA) was used to estimate insulin resistance (HOMA-IR) in the fasting state and was calculated as follows: FPG [mmol/L] × fasting insulin [U/L]/22.5. Elevated insulin resistance was defined as a HOMA-IR > 2.5. As index of β-cell function, HOMA-β-cell was calculated as 20× fasting insulin [U/L]/FPG [mmol/L] − 3.5 [35].

### 2.5. Biochemical Analyses

Glucose, insulin, and peptide C concentrations were determined in plasma (for glucose) or in serum (for insulin and C-peptide) at 0 (T0), 10 (T1), 20 (T2), 30 (T3), 60 (T4), 90 (T5), 120 (T6), 150 (T7), and 180 (T8) minutes following a standard dose of 75-g glucose load. 

### 2.6. Arg/NO Metabolic Pathway

Arg, asymmetric dimethylarginine (ADMA), symmetric dimethylarginine (SDMA), citrulline (Cit) and ornithine (Orn) were simultaneously measured by liquid chromatography—tandem mass spectrometry (LC-MS/MS) both in plasma and in RBCs [36]. The ratio Arg/(Orn + Cit), as an index of global Arg availability [37,38] and the ratio Orn/Cit, as an index of the relative activity of arginase and NOS [39], were computed. 

### 2.7. Oxidative Stress 

Urine samples were collected after overnight fasting and at 180 min after the oral glucose load of the OGTTs. The samples were stored at −80°C until analysis.

Urinary 8-iso-PGF_2α_ was detected by LC-MS/MS method according to Cavalca et al. [40]. The urinary concentration was calculated from the area ratio of the ion peaks of the 8-iso-PGF_2α_ over the respective deuterated standard (8-iso-PGF_2α_-d_4_). The analyte estimated value was corrected for the urinary creatinine level and expressed as pg/mg creatinine. Reduced glutathione (GSH) and disulphide glutathione (GSSG) were assessed by LC-MS/MS method in RBCs after protein precipitation with trichloroacetic acid [41]. Levels of GSH and GSSG are expressed as µmol/g Hb. 

### 2.8. Nitric Oxide Synthase Analysis in Red Blood Cells 

The RBC-NOS expression was evaluated by immunofluorescence analysis. RBCs were dispersed on a slide and heat-fixed as previously described [33]. After blocking of non-specific reactive sites, the slides were incubated overnight at 4 °C with a polyclonal anti-eNOS type III (1:250) antibody (USBiological, Società Italiana Chimici, Rome, Italy), specific for the translocated and active form of eNOS [42]. Detection was performed with anti-rabbit AlexaFluor488 conjugated secondary antibody (1:100) (Invitrogen, Life Technologies Italia, Monza, Italy), and the immune complexes were visualized by laser scanning confocal microscope (LSM710, Zeiss, Milano, Italy) using a 63×/1.3 oil immersion objective lens. Fluorescent images were captured with a digital camera. Data are expressed as the mean level of fluorescence intensity, subtracted from negative control value obtained in the absence of primary antibody. Multiple fields of view (at least three randomly selected areas) were captured for each slide.

### 2.9. Phosphatidylserine Exposure 

After precipitation, RBCs (1 × 10^6^ cell/mL) were resuspended in annexin V-binding buffer and stained with PE Annexin-V (BD Pharmingen, Milan, Italy). After 20 min samples were analyzed by flow cytometer FACS-Calibur (BD Biosciences, Milan, Italy). Forward and side scatter light was used to identify cell population and annexin V-fluorescent intensity was measured in the FL-2 channel in 50,000 events.

### 2.10. Statistical Analysis

Continuous variables were expressed as mean ± standard deviation (SD) or median with interquartile range (IQR), according to their distribution, while categorical variables were shown as absolute numbers and percentages. Paired T-test or Wilcoxon signed-rank test was applied two evaluate difference between baseline and time specific measure. Normality of the data was assessed through graphical methods (such as histograms and qq-plot) and tests (Kolmogorov-Smirnov). Correlation between variables were assessed using Spearman’s Test. All tests were 2-tailed and a *p* < 0.05 was considered statistically significant. All analyses were performed using SAS version 9.4 (SAS Institute, Cary, NC, USA).

## 3. Results

### 3.1. Characteristics of the Study Population

The demographic and clinical characteristics of patients participating in the study are summarized in Table 1. Patients were slightly overweight as shown by the BMI values. The systolic/diastolic pressure values and the lipid profile were within the normal reference range, in most cases as a result of pharmacological treatments.

Data are expressed as mean ± SD or median and interquartile range. BMI: body mass index; LDL: low density lipoprotein; HDL: high density lipoprotein; HOMA-IR: homeostasis model assessment as index of insulin resistance; HOMA-β-cell: homeostasis model method of assessment β-cell function.

**Figure 1:**
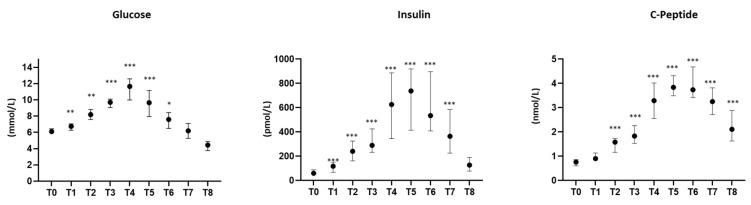
Levels of glucose, insulin, and C-peptide at baseline (T0) and for 180 min following an oral glucose load in prediabetic patients. Data are expressed as median and interquartile range. *n* = 18; * *p* < 0.05, ** *p* < 0.005, *** *p* < 0.0005 vs. T0.

Figure 1 shows the circulating levels of glucose, insulin, and C-peptide, measured in prediabetic patients during OGTT. A significant increase in glucose levels was detected starting 10 min after glucose load (T1), with the peak at T4. The insulin levels significantly increased at T1 with a peak at T5 and, in accordance with insulin levels, peptide C that is co-secreted with insulin from pancreatic β-cells also progressively increased with a peak at T5.

### 3.2. Oxidative Stress Status

**Figure 2:**
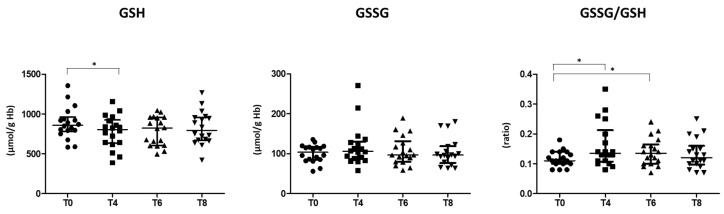
Levels of reduced glutathione (GSH), disulphide glutathione (GSSG), and the ratio GSSG/GSH at baseline (T0) and at the indicated times following an oral glucose load in RBCs isolated from prediabetic patients. ● T0; **■** T4; ▲ T6; **▼** T8. Data are expressed as median and interquartile range. *n* = 18; * *p* < 0.05.

In prediabetic patients, OGTT did not induce a burst of systemic oxidative stress within 3 h after glucose loading, as documented by unchanged urinary levels of 8-iso-PGF2α (154.1 ± 52.4 and 156.0 ± 59.1 pg/mg creatinine, at T0 and T8 respectively; *p* = 0.880). In contrast, a significant increase in RBCs oxidative status was evidenced by the rising of the GSSG/GSH ratio (Figure 2), mainly due to a significant reduction in the GSH content at T4 and T6. 

Moreover, a positive correlation between GSSG and glucose variation levels in the period T0–T4 was evidenced (Spearman’s correlation: r = 0.504, *p* = 0.033), also adjusting for age and BMI (Spearman’s partial correlation: r = 0.515, *p* = 0.041). 

### 3.3. Arg/NO Pathway in RBCs of Prediabetic Patients

**Figure 3:**
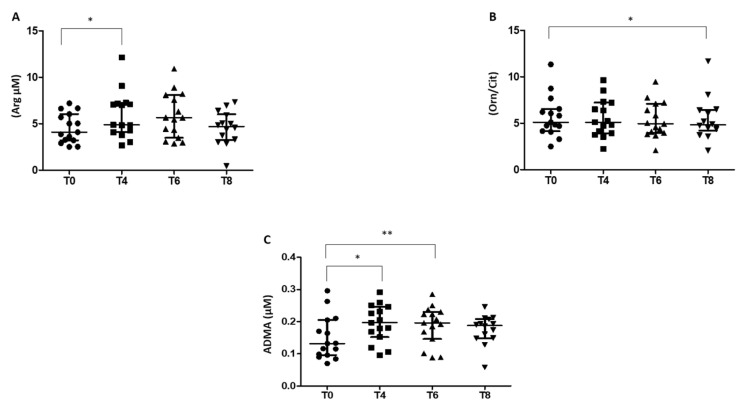
Levels of (**A**) arginine (Arg), (**B**) ratio ornithine and citrulline (Orn/Cit), and (**C**) asymmetric dimethylarginine (ADMA) at baseline (T0) and for 180 min following an oral glucose load in RBCs isolated from prediabetic patients. ● T0; **■** T4; ▲ T6; **▼** T8. Data are expressed as median and interquartile range. *n* = 18; * *p* < 0.05, ** *p* < 0.005.

The glucose load altered the Arg/NO pathway in RBCs, with an increase of the NO precursor Arg evidenced at T4 (Figure 3A), which progressed in parallel with a significant reduction of Arg in the plasma compartment (80.97 ± 17.99 and 72.51 ± 14.38 at T0 and T4 respectively, *p* = 0.01). The increase of Arg levels in RBCs at 60 min after glucose load (T4) was not accompanied by significant variations in the levels of the two metabolites Cit and Orn generated from NOS and arginase activity, respectively. In contrast, the Orn/Cit ratio, an indirect index of arginase and NOS activities, showed a slight decrease from T0 to T4, with a significant reduction 180 min after OGTT (Figure 3B). 

Furthermore, in RBCs, the levels of the endogenous inhibitor of NO formation, ADMA, showed a similar trend to that of Arg (Figure 3C).

In contrast, no difference was detected after OGTT in the levels of SDMA, a competitor of arginine transport (0.10 ± 0.04, 0.11 ± 0.03, 0.12 ± 0.03, 0.11 ± 0.04, at T0, T4, T6, and T8, respectively). Together, these results highlight a reduction in NO production in RBCs during OGTT until 120 min after glucose load (T6), followed by an increase at 180 min (T8). 

**Figure 4:**
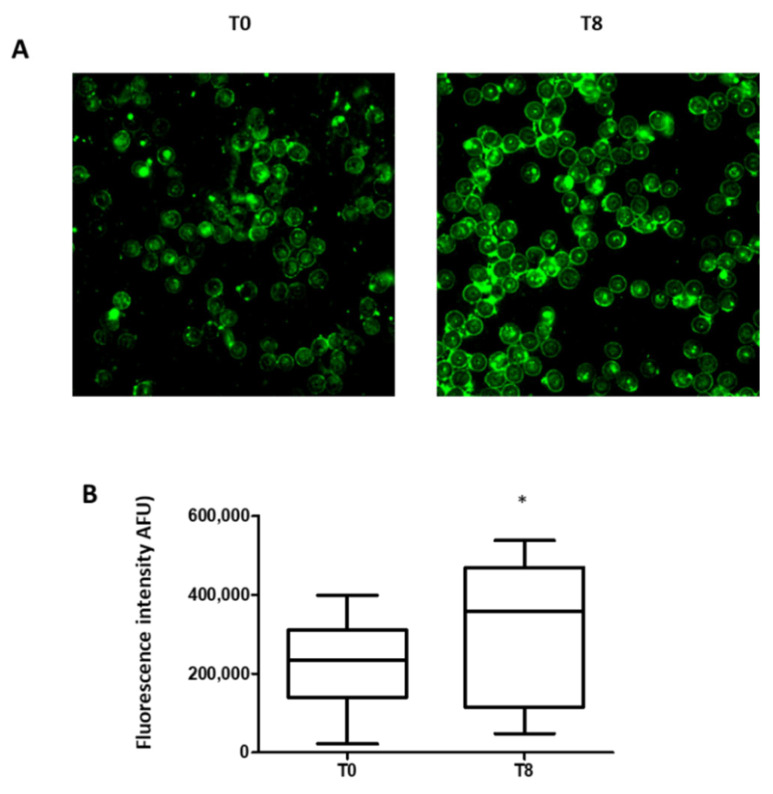
NO synthase expression. (**A**) Representative immunofluorescent images (630× magnification) of RBCs isolated from prediabetic patients stained for RBC-NOS at baseline (T0) and at 180 min after an oral glucose load. (**B**) Quantitative analysis of RBC-NOS. Data are expressed as the mean of fluorescent intensity (AFU) ± SD subtracted of the negative control value (at least three fields were analyzed). 𝑛 = 13; * *p* < 0.05.

Further, the expression of the activated RBC-NOS, detected by immunofluorescence, was significantly increased 180 min after OGTT (T8) compared to T0 (Figure 4A,B). 

### 3.4. Phosphatidylserine Exposure in RBCs

**Figure 5:**
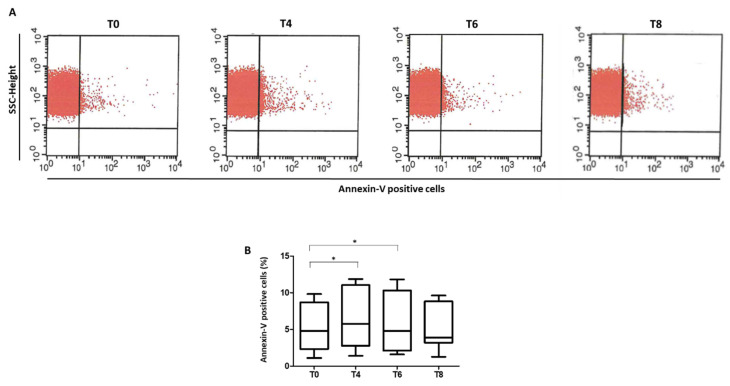
Annexin V positive RBCs. RBCs were analyzed by flow cytometry after staining with PE-conjugated annexin V. (**A**) Representative dot-plot graph of RBCs isolated from prediabetic patients. (**B**) Quantitative analysis of annexin V RBCs. Data are expressed as percent of positive RBCs ± SD. 𝑛 = 11; * *p* < 0.05.

The exposure of phosphatidylserine was measured in RBCs of patients during OGTT at T0, 60 (T4), 120 (T6), and 180 (T8) minutes after glucose loading. The glucose load increased the percentage of annexin V-binding RBCs in prediabetic patients. This increment was significant at T4 and T6 compared to baseline. In contrast, the annexin V positive cells at T8 were similar to that detected at T0 (Figure 5A,B). Moreover, a borderline positive correlation between the annexin V positive RBCs at T4 and GSSG levels was found (Spearman’s correlation: r = 0.582, *p* = 0.060). 

### 3.5. Red Cell Distribution Width 

The red cell distribution width (RDW), a measure of the variability of volume, has been recently considered an inflammatory marker and predictor of mortality and adverse outcomes in several cardiovascular diseases [43]. In our population we evidenced a positive correlation between RDW-SD and glucose levels at 180 min after glucose load (Spearman’s correlation r = 0.521, *p* = 0.031), also adjusting for age and BMI (Spearman’s partial correlation r = 0.513, *p* = 0.051). 

## 4. Discussion

In this study, we analyzed oxidative stress status, Arg/NO metabolic pathway, and PS exposure in RBCs isolated from patients with prediabetes during a 75 g-OGTT. Our results show a marked increase in oxidative stress levels 60 min after glucose loading, in parallel with a decreased NO production up to 120 min. These metabolic alterations rescue 180 min after OGTT, alongside an increase in activated RBC NOS expression.

Postprandial hyperglycemia is an independent risk factor for cardiovascular disease in patients with diabetes or prediabetes and it has been shown that glucose levels 2 h after OGTT is a better predictor of coronary heart disease and mortality than fasting glucose [8,44]. In particular, this high sensitivity has been shown in subjects with an apparent good glycemic control but with an impaired response to the oral glucose loading in terms of magnitude and slope of the hyperglycemic peaks [45]. An acute increase of glycemia directly enhances several risk-related pathways such as LDL oxidation [46], endothelial dysfunction [30], activation of the coagulation cascade [47], and expression of adhesion molecules [48]. 

### 4.1. Oxidative Stress Status

Although the mechanisms by which postprandial hyperglycemia induces an increase of vascular complications are still not well defined, it is known that acute hyperglycemia induces the generation of reactive oxygen species, resulting in an increase of oxidative stress [49]. To date, information on the relationship between a transient and acute increase in plasma glucose levels and oxidative stress is contrasting [17,30,50,51,52]. These previous contradictory findings could depend on different analytical techniques used and/or the type of oxidative stress marker chosen. Indeed, the presence of oxidative stress can be evidenced through direct measurements of reactive oxygen species, employing techniques such as electron spin resonance spectroscopy, or through the evaluation of antioxidant defense systems, and each methodology presents advantages and disadvantages [53,54]. 

Oxidative stress is triggered by an increase in the generation of intracellular and extracellular free radicals. GSH is an important ubiquitous antioxidant and detoxifier of free radicals that plays a pivotal role in cellular protection, and its ratio with the oxidized form GSSG is used as a marker of oxidative stress [55]. In our study, we showed an increase of oxidative stress evidenced by the increase of the GSSG/GSH ratio at 60 min after the glucose loading, which lasted until 90 min. One hundred and eighty minutes after the oral glucose load the GSSG/GSH ratio was similar to that detected at baseline. Overall, these results highlight a parallel trend between oxidative stress induced by acute hyperglycemia and glucose levels. In accordance, a positive correlation between the increase in the glucose levels in the range T0-T4 and GSSG levels was also evidenced.

### 4.2. Arginine/Nitric Oxide Pathway 

It has been shown that following glucose loading, acute hyperglycemia reduced the endothelial function detected by the measurement of flow-mediated dilatation of the brachial artery, both in subjects with normal and with IGT [31,56]. The authors suggested that this endothelial dysfunction is mediated by a decrease of NO production [49]. In accordance, our data show a significant increase of the Orn/Cit ratio in plasma (1.96 ± 0.44 and 2.52 ± 0.67 at T0 and T4 respectively, *p* = 0.0002), suggesting an increase in arginase activity. In contrast, in RBC the glucose loading leads to a progressive reduction of Orn/Cit ratio that becomes statistically significant 180 min after OGTT, when a marked increase of activated RBC-NOS was observed. Moreover, at T4 and T6, we found a significant increase in the levels of ADMA, the competitive inhibitor of NOS. In contrast, at T8 the ADMA levels were similar to that measured at T0. An increase of ADMA following OGTT has been shown both in subjects with normal glycemia and with impaired glucose tolerance [48] and high levels of ADMA have been detected not only in patients with diabetes, but also in prediabetics [57]. In this context, it has been suggested that the high ADMA levels may be an epiphenomenon associated with high glucose levels, a documented cause for the increase of ADMA via inhibition of intracellular oxidative stress-dependent dimethylarginine dimethylaminohydrolase activity [58].

The enzymatic process to produce NO requires oxygen and several cofactors including calcium, calmodulin, NADPH, FMN, FAD, and tetrahydrobiopterin. In particular, it has been shown that the interaction of calmodulin, the small cytosolic calcium-binding protein, is able to induce a conformational change in NOS resulting in the activation of the enzyme [59]. Nevertheless, this issue was not addressed in our study, and deserves further investigation. 

### 4.3. Eryptotic Process

The capability of RBC to produce NO represents an important for its functions, and in vitro and in vivo studies have evidenced a role of RBC-NOS in modulating erythrocrine functions [34,60,61]. Although the RBC-NOS does not seem to affect the erythrocyte membrane fragility [62], a reduction of its activity might affect the RBC lifespan [34].

The lifespan of circulating erythrocytes is about 120 days, after which they are removed from circulation by the process of senescence [63,64]. However, under certain conditions, before senescence, RBCs may undergo a suicidal death, namely eryptosis, characterized by the exposure of PS on the cell membrane [65]. It is known that in diabetes the lifespan of erythrocytes is reduced and eryptosis is enhanced [23,24]. The increase in eryptosis is, at least in part, dependent on an accumulation of methylglyoxal, an extra- and intra-cellular metabolite formed as a by-product of glucose metabolism, able to reduce GSH levels in erythrocyte and to induce PS exposure [28]. In accordance, we found an increased exposure of PS on RBCs membranes at T4 and T6 and, in parallel, a marked decrease of GSH at T4. Supporting this result, a positive correlation between GSSG levels and PS exposure was found, suggesting that the increase of annexin V positive RBCs may be related to an increase in oxidative stress.

Blebbing of RBC membrane during eryptosis could lead to the release of microvesicles into the blood circulation [66]. An increase in erythrocyte-derived microvesicles has been associated with endothelial dysfunction and increased levels of microvesicles have been detected in several cardiovascular diseases, including diabetes [67]. However, whether erythrocyte-derived microvesicles contribute to the diabetes-related complications or disease progression needs to be further investigated.

### 4.4. Red Cell Distribution Width 

Finally, in our population, a positive correlation was evidenced between glucose levels at 180 min and RDW, a powerful predictor of mortality, and other adverse outcomes in several cardiovascular settings [68,69,70]. Accordingly, Tripolino and colleagues found that RDW associates with plasma glucose concentrations after 75 g-OGTT [71]. Even if the mechanisms underlying this association are not well defined yet, oxidative stress and inflammation could play a role in this context [72]. 

### 4.5. Limitations of the Study

This study has some limitations. The number of patients studied was small and the enrolled subjects were all men. These conditions may limit the general applicability of our findings, and care must be taken when extrapolating our results to women. In addition, most of the subjects enrolled were pharmacologically treated with statins (61%), aspirin (61%), and calcium blockers (44%); therefore, we cannot rule out the impact of these drugs on the studied phenomena. In addition, measurement of glycated hemoglobin, another test that can be used for the diagnosis of diabetes, was not performed in this study. 

It is known that obesity is associated with changes in RBC metabolism. Although differences in arginine and citrulline levels have been shown in RBCs obtained from obese (BMI > 30 Kg/m^2^) compared to normal BMI subjects [73], we cannot exclude that in our population, subjects with BMI > 25 also show alterations in oxidative stress and NO pathway. Finally, the study design did not include a control group, due to ethical reasons. Nevertheless, all samples obtained from a subject were analyzed in the same analysis in order to avoid any technical bias. 

## 5. Conclusions

In conclusion, our study shows that a 75-g oral glucose load in patients with prediabetes is associated with oxidative stress, inhibition of NO metabolic pathway, and increase in eryptosis in RBCs. In particular, these events have been evidenced up to 60 min after the glucose load, the time at which we detected the maximum peak of circulating glucose. After this time, the decrease of plasma glucose levels progresses in parallel with a reduction of oxidative stress, recovery of the active form of RBC-NOS, and decrease of the annexin V positive RBCs. Overall our results support the relationship between the increase of glycaemia and oxidative stress, and highlight the impairment of the Arg/NO pathway and the exposure of PS on RBC membrane, all features detrimental for the cardiovascular system. Therefore, the results of this study provide a possible explanation to some phenomena observed after glucose loading and suggest that even in the early stages of diabetes, it may be important to avoid acute variations to prevent diabetic complications. 

## Figures and Tables

**Table 1:** Baseline demographic and clinical characteristics of the study population.

Variables	Prediabetic Patients (*n* = 18)
Age (years)	64.1 ± 5.6
Male sex, *n* (%)	18 (100)
BMI (kg/m^2^)	26.3 ± 2.6
Abdominal circumference (cm)	93.6 ± 9.81
Central obesity, *n* (%)	3 (17)
Metabolic syndrome, *n* (%)	11 (61)
Systolic blood pressure (mmHg)	129 ± 10
Diastolic blood pressure (mmHg)	75 ± 8
Total cholesterol (mmol/L)	4.3 ± 1.0
LDL cholesterol (mmol/L)	2.7 ± 0.9
HDL cholesterol (mmol/L)	1.1 ± 0.3
Triglycerides (mmol/L)	1.1 ± 0.5
Creatinine (μmol/L)	77 ± 14
HOMA-IR	2.68 ± 1.00
HOMA-β-cell	66.69 (47.6; 94.14)
RDW-SD (fL)	40.9 ± 2.9
**Pharmacological treatments**	
Aspirin, *n* (%)	11 (61)
Beta-blockers, *n* (%)	2 (11)
Calcium blockers, *n* (%)	8 (44)
Angiotensin converting enzyme inhibitors, *n* (%)	2 (11)
Angiotensin II receptor antagonists, *n* (%)	4 (22)
Statins, *n* (%)	11 (61)

## Data Availability

Data collected in the study will be made available using the data repository Zenodo (https://zenodo.org (accessed on 11 June 2022) with restricted access upon request to direzione.scientifica@ccfm.it (accessed on 11 June 2022).
